# Six Additions to the Genus *Periconia* (Dothideomycetes: Periconiaceae) from Graminaceous Plants in China

**DOI:** 10.3390/jof9030300

**Published:** 2023-02-25

**Authors:** Pengwei Su, Zhenghua Lu, Whenhui Tian, Yanpeng Chen, Sajeewa S. N. Maharachchikumbura

**Affiliations:** School of Life Science and Technology, Center for Informational Biology, University of Electronic Science and Technology of China, Chengdu 611731, China

**Keywords:** 5 new records, 6 new species, Ascomycota, fungal taxonomy, multi-locus phylogeny, Sichuan Province

## Abstract

*Periconia* is a polyphyletic and asexual morphic genus within the family Periconiaceae (Pleosporales). The genus is characterized by a pale to dark brown stipe with an apical conidial head and ellipsoidal to oblong conidia. Species of *Periconia* are widely distributed throughout the world in various hosts, while most species are isolated from graminaceous plants. During our investigations of microfungal in Sichuan Province, China, 26 *Periconia* isolates were collected from a wide variety of graminaceous plants. These isolates corresponded to 11 species based on the examination of morphology and multi-locus phylogenetic analysis (SSU, ITS, LSU, *TEF1*, *RPB2*). This includes six new species (*P. chengduensis*, *P. cynodontis*, *P. festucae*, *P. imperatae*, *P. penniseti,* and *P. spodiopogonis*) and five new records (*P. byssoides*, *P. chimonanthi*, *P. cookie*, *P. pseudobyssoides*, and *P. verrucosa*). A comprehensive description and illustrations of the new species are provided and discussed with comparable taxa. These discoveries expand our knowledge of the species diversity of *Periconia* taxa in graminaceous plants in China.

## 1. Introduction

The genus *Periconia* (Periconiaceae, Pleosporales) was introduced by H.J. Tode, with *P. lichenoides* as the type species [1]. Most *Periconia* species are mainly known from their asexual morphs, characterized by conidiophores that are macronematous, mononematous, branched or unbranched, and pale to dark brown [2,3], while conidiogenous cells are discrete on the terminal or intercalary of the stipe and are monoblastic to polyblastic [2,4]. Conidia of *Periconia* species are globose to ellipsoidal, catenate or solitary, smooth or verruculose, and pale brown to brown [2,4,5,6]. Only five species have been reported to have sexual morphs, viz., *P. didymosporum*, *P. homothallica*, *P. igniaria*, *P. prolifica,* and *P. pseudodigitata* [7,8]. The sexual morph is characterized by scattered or grouped, globose ascomata with a central ostiole, hyaline periphyses, 8-spored asci, and broadly fusiform, 1-septate, hyaline and smooth ascospores with an entire sheath [7,8,9,10]. *Periconia* species are widely distributed and usually found in terrestrial habitats and rarely in aquatic habitats [11,12]. The genus comprises many saprophytes and endophytes, while few are plant pathogens, mainly causing diseases in graminaceous plants [2,4,13,14,15]. For example, *P. circinata* causes the blackening and rotting of wheat roots and stem bases (milo disease), and *P. macrospinosa* causes leaf necrosis in the pointed gourd (Cucurbitaceae) [16,17]. Gunasekaran et al. [18] reported that *P. atra* causes human corneal ulcers. Several species, such as *P. atropurpurea* and *P. siamensis,* are known to produce aromatic compounds and macrolide compounds with tremendous pharmacological activities [19,20]. 

*Periconia* is a polyphyletic genus in the family Periconiaceae [21]. The members of this genus were previously classified under Massarinaceae. Based on phylogenetic analysis, Tanaka et al. [7] showed that Periconiaceae is a sister clade distinct from Massarinaceae [22]. Phukhamsakda et al. [23] showed that Periconiaceae and Massarinaceae diverged in the late Cretaceous period (around 70 million years ago). The family Periconiaceae includes four genera: *Bambusistroma*, *Flavomyces*, *Noosia*, and *Periconia*. The genus *Periconia* has 211 epithets in Index Fungorum (http://www.indexfungorum.org/; accessed on 20 January 2023). Among these, 29 species have been transferred to other genera [24,25,26,27,28,29,30], and 7 species have been synonymized under other *Periconia* species [2,31,32,33]. In the previous three years, nine *Periconia* species have been introduced [6,8,34,35,36,37]. There are 124 accepted *Periconia* species in Species Fungorum, but only 37 species have sequence data.

In this study, 26 *Periconia* isolates were obtained from several collection sites from July to October 2021. Based on morphological studies and multi-locus phylogenetic analysis, these isolates were assigned to 11 species, including 6 new species and 5 new records. This study aims to describe these new taxa with detailed descriptions and illustrations and to broaden our understanding of the diversity of periconia-like taxa.

## 2. Materials and Methods

### 2.1. Sample Collection, Morphological Examination, Isolation, and Preservation

A survey on a fungal diversity of hyphomycetes fungi on graminaceous plants in Sichuan Province, China, was conducted from July to October 2021 at five natural sites in Sichuan Province (Huilonggou, Pengzhou City; Guoxue Park, Chengdu; Longchi National Forest Park, Chengdu; Baiyungou, Chunzhou City; Xiqiang Valley, Wenchuan County, Ngawa Tibetan and Qiang Autonomous Prefecture). These specimens were stored in paper envelopes and returned to the laboratory for examination. The morphological observation was carried out from material on natural substrates using a Motic SMZ 168 series stereomicroscope. The mycelia were placed in sterile water for micromorphological observation, and the fungal microstructures were photographed using the DS-Fi3 camera fitted with a Nikon Eclipse Ni-U microscope. All measurements were conducted using Nikon NIS-Elements D 5.21 software, and the photos were processed using Adobe Photoshop version 21.0. Pure cultures were obtained by single spore isolation according to the method described by Senanayake et al. [38]. The germinated conidia were then transferred individually to potato dextrose agar (PDA) plates and incubated in the dark at 25 °C. Culture characteristics were examined and recorded after one week and later at regular intervals.

The specimens were deposited in the Herbarium of Cryptogams Kunming Institute of Botany Academia Sinica (HKAS), Kunming, China, or the Herbarium of the University of Electronic Science and Technology (HUEST), Chengdu, China. The living cultures were deposited in the China General Microbiological Culture Collection Center (CGMCC), Beijing, China, and the University of Electronic Science and Technology Culture Collection (UESTCC), Chengdu, China. The taxonomic descriptions of the new taxa have been deposited with MycoBank (https://www.mycobank.org/; accessed on 1 February 2023).

### 2.2. DNA Extraction, PCR Amplification, and Sequencing

Fungal genomic DNA was extracted from mycelia using the Trelief^TM^ Plant Genomic DNA Kit (TSINGKE Biotech, Shanghai, China) according to the manufacturer’s protocol. The DNA was stored at −20 °C for long-term storage. The five loci, which include the nuclear ribosomal internal transcribed spacer (ITS: ITS1-5.8S-ITS2), the partial nuclear ribosomal small subunit rRNA (SSU), the partial nuclear ribosomal large subunit rRNA (LSU), the partial translation elongation factor 1-alpha (*TEF1*), and the partial second-largest subunit of RNA polymerase II (*RPB2*), were amplified by polymerase chain reaction (PCR). The corresponding primer pairs and PCR conditions are listed in Table 1. The final PCR reaction system was 25 µL, containing 12.5 μL PCR Master Mix (Sangon Biotech, Shanghai, China), 8.5 µL of double-distilled water (ddH_2_O), 1 μL each of forward and reverse primers, and 2 μL DNA template. The PCR products were visualized by electrophoresis in 1% agarose gels. Sanger sequencing was conducted by Tsingke Biological Technology (Beijing, China).

### 2.3. Phylogenetic Analyses

The raw sequencing fragments of corresponding Sanger sequencing chromatograms were manually edited, trimmed, and assembled into consensus sequences using SeqMan Pro version 11.1.0 (DNASTAR, Inc. Madison, WI, USA). Barcode sequences of *Periconia* species currently available in GenBank and the outgroup taxon *Massarina cisti* (CBS 266.62) were downloaded from the NCBI nucleotide database using an in-house python script.

The multiple sequence alignment was conducted using MAFFT version 7.310 [46] with options “--maxiterate 1000 --genafpair --adjustdirectionaccurately”, and the alignment results were further trimmed using trimAl version 1.4 [47] with the option “-gapthreshold 0.5”, which only allows 50% of taxa with a gap in each site. The best-fit nucleotide substitution models for each alignment dataset were selected using PartitionFinder version 2.1.1 [48] under the Akaike Information Criterion (AIC).

Maximum likelihood (ML) and Bayesian analysis (BI) were conducted based on the individual and combined datasets. ML phylogenetic trees were obtained using the IQ-TREE version 2.0.3 [49], and the topology was evaluated using 1000 ultrafast bootstrap replicates. The BI was conducted using parallel MrBayes version 3.2.7a [50]. Two different runs with 20 million generations and four chains were executed, and the initial 25% of sample trees were treated as burn-in. Tracer version 1.7.1 [51] was used to confirm that the MCMC runs reached convergence with all ESS values above 200. Then, the ML tree was annotated by TreeAnnotator version 2.6.6 implemented in BEAST version 2.6.6 [52] based on MrBayes MCMC trees with no discard of burn-in and no posterior probability limit. The tree was visualized using ggtree [53] and edited in Adobe Illustrator version 20.0.0.

## 3. Results

### 3.1. Molecular Phylogeny

The newly generated sequences were deposited in GenBank, and the accession numbers were listed in Table 2. The combined dataset included five loci, SSU, ITS, LSU, *TEF1,* and *RPB2,* from 82 isolates of *Periconia* with *Massarina cisti* (CBS 266.62) as the outgroup taxon. The concatenated alignment comprises 4320 characters (SSU: 1–1001; ITS: 1002–1552; LSU: 1553–2411; *TEF1*: 2412–3309; *RPB2*: 3310–4320), including gaps, consisting of 1389 distinct patterns, 804 parsimony-informative, 330 singleton sites, and 3186 constant sites. Five single-locus datasets, SSU, ITS, LSU, *TEF1,* and *RPB2,* contained 30, 240, 91, 123, and 320 parsimony-informative sites, respectively. The best-fit evolution models were GTR+I+G for the SSU, ITS, LSU, *TEF1*, and *RPB2*.

**Figure 1:**
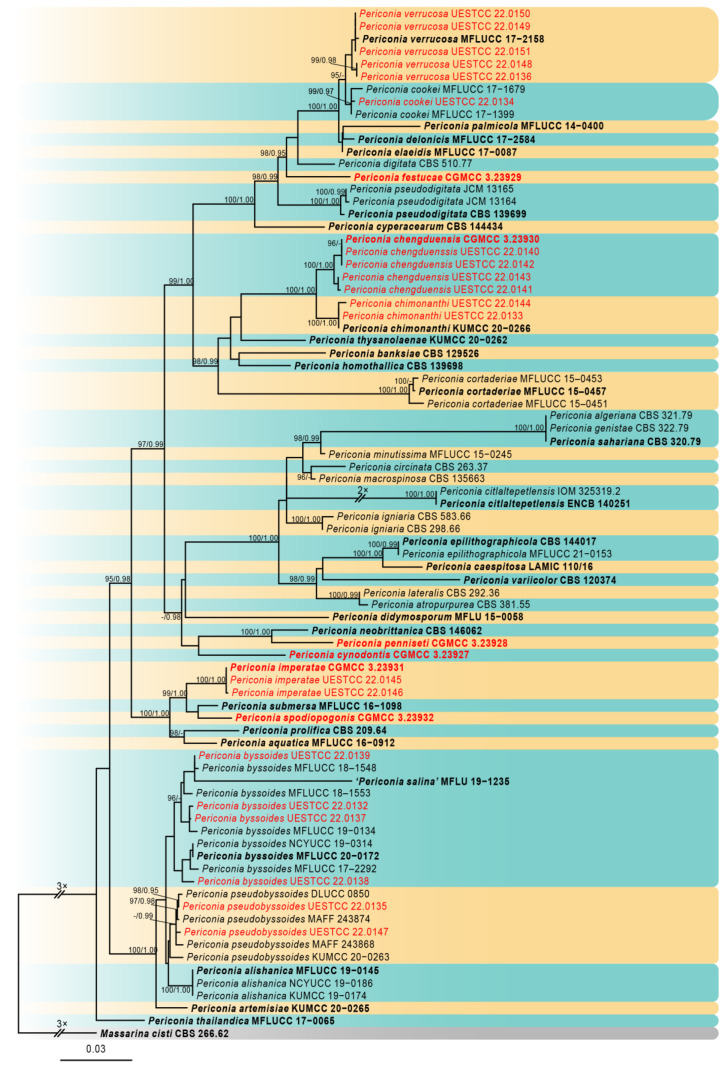
Phylogram of the best-scoring ML consensus tree based on a combined dataset (SSU, ITS, LSU, *TEF1,* and *RPB2*) of *Periconia*. Novel isolates are indicated in red. Isolates from type materials are in bold. The ML ultrafast bootstrap values/Bayesian PP greater than 95%/0.95 are shown at the respective nodes. The tree is rooted with *Massarina cisti* CBS 266.62 (Massarinaceae, Pleosporales).

The best-scoring ML consensus tree (lnL = −20,240.300) with ultrafast bootstrap values from ML analyses and posterior probabilities from MrBayes analysis at the node is shown in Figure 1. Phylogenetic analyses showed that our newly collected 26 isolates clustered into 11 clades and can be recognized as 5 known species (*P. byssoides*, *P. chimonanthi*, *P. cookie*, *P. pseudobyssoides,* and *P. verrucosa*) and six new species (*P. chengduensis*, *P. cynodontis*, *P. festucae*, *P. imperatae*, *P. penniseti,* and *P. spodiopogonis*).

### 3.2. Taxonomy

***Periconia byssoides*** Pers., Syn. meth. fung. (Göttingen) 2: 686 (1801) Figure 2.

*MycoBank*: MB 144538.

*Saprobic* on dead culms of Poaceae. **Asexual morph**: *Colonies* on the natural substrate numerous, effuse, brown to dark brown, hairy. *Conidiophores* 240–410 μm long ($\bar{x}$ = 354, *n* = 15), 12–21 μm wide ($\bar{x}$ = 17 μm, *n* = 15), macronematous, mononematous, straight or slightly flexuous, unbranched, solitary, rarely 1–2 together on stroma, brown to dark brown, 2–4-septate (majority 3-septate), smooth to minutely verruculose, thick-walled. *Conidiogenous cells* polyblastic, pale brown to brown, subglobose, smooth or verruculose. *Conidia* 11.5–18.5 × 12–18 μm ($\bar{x}$ = 15 × 14.5 μm, *n* = 40), solitary or catenate, globose, brown, aseptate, echinulate, or verruculose. **Sexual morph**: Undetermined.

*Materials examined*: China, Sichuan Province, Ngawa Tibetan and Qiang Autonomous Prefecture, Wenchuan County, Xiqiang Valley, 31°29′27″ N, 103°37′1″ E, elevation 1500 m, on dead culms of *Imperata cylindrica* (Poaceae), 20 October 2021, ZH Lu w281_1 (HUEST 22.0133), living culture UESTCC 22.0132; *ibid*., w282 (HUEST 22.0140), living culture UESTCC 22.0139; Chongzhou City, Baiyungou, 30°47′35″ N, 103°23′49″ E, elevation 990 m, on dead culms of *Imperata cylindrica* (Poaceae), 27 September 2021, ZH Lu Lu44 (HUEST 22.0138), living culture UESTCC 22.0137; *ibid*., Lu50 (HUEST 22.0139), living culture UESTCC 22.0138.

*Culture characteristics*: Colony on PDA reaching 50 mm diam after one week in an incubator under dark conditions at 20 °C, circular, cottony, hairy at the margin, white; pale yellow at the margin and mud yellow at the middle in reverse.

*Notes*: The phylogenetic tree showed that our four isolates (UESTCC 22.0132, UESTCC 22.0137, UESTCC 22.0138, UESTCC 22.0139) grouped with other *P. byssoides* isolates, including the type (MFLUCC 20–0172). Our collections (HUEST 22.0133, HUEST 22.0138, HUEST 22.0139, HUEST 22.0140) share similar morphological characteristics in shape and color of conidiophores and conidia with the type of *P. byssoides*. Therefore, we identified our new isolates as *P. byssoides* based on the overlapping morphological characteristics and the multi-locus phylogenetic tree, and one collection (HUEST 22.0133) is a new host record from *Imperata cylindrica*. 

**Figure 3:**
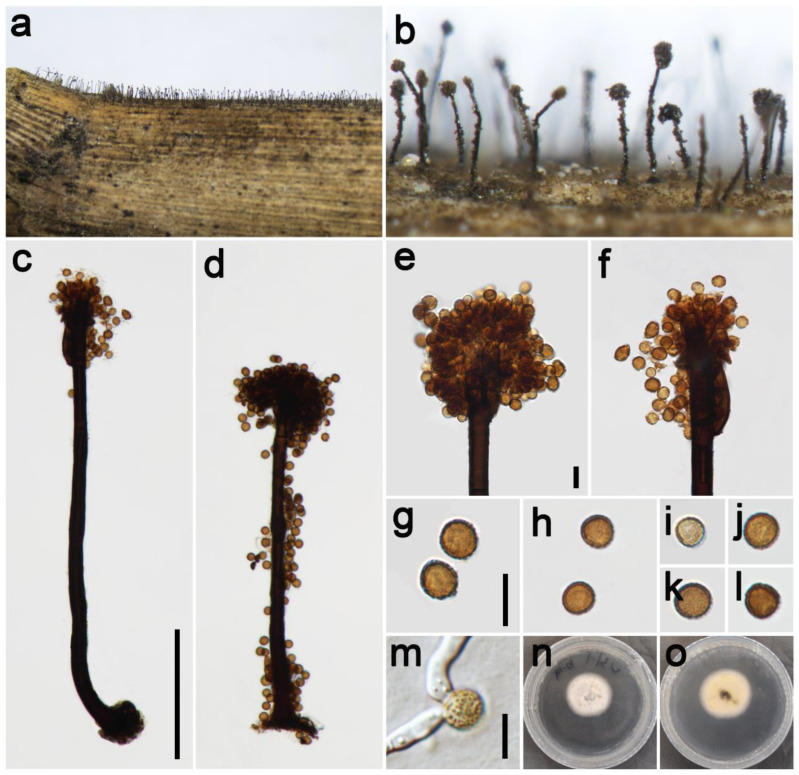
*Periconia chengduensis* (HKAS 126514, holotype). (**a**,**b**) Colonies on the natural substrate; (**c**,**d**) conidiophores with spherical conidial heads; (**e**,**f**) apically branch conidiophores with conidial head; (**g**–**l**) conidia; (**m**) germinating conidium; (**n**,**o**) colony on PDA from above and below. Scale bars: (**c**) = 100 μm, (**e**,**g**,**m**) = 10 μm. Scale bar of (**c**) applies to (**d**). Scale bar of (**e**) applies to (**f**). Scale bar of (**g**) applies to (**h**–**l**).

***Periconia chengduensis*** Z.H. Lu, P.W. Su, and Maharachch., sp. nov. Figure 3.

*MycoBank*: MB 847458

*Etymology*: Name refers to Chengdu, the city where the fungus was collected.

*Saprobic* on dead culms of Poaceae. **Asexual morph**: *Colonies* on the natural substrate numerous, effuse, dark brown to black, hairy. *Conidiophores* 240–370 μm long ($\bar{x}$ = 292, *n* = 15), 10–13 μm wide ($\bar{x}$ = 12 μm, *n* = 15), macronematous, mononematous, straight or slightly flexuous, branched, solitary, rarely 1–2 together on stroma, dark brown to black, septate, smooth to minutely verruculose, thick-walled. *Conidiogenous cells* polyblastic, pale brown to brown, terminal, integrated, subglobose, and smooth to verruculose. *Conidia* 5–8.5 × 5–8 μm ($\bar{x}$ = 7.2 × 7 μm, *n* = 40), solitary or catenate, oval to globose, brown to dark brown, aseptate, echinulate or verruculose. **Sexual morph**: Undetermined.

*Materials examined*: China, Sichuan Province, Chengdu City, Longchi National Forest Park, 31°06′15″ N, 103°33′32″ E, elevation 2000 m, on dead culms of *Pennisetum purpureum* (Poaceae), 19 September 2021, ZH Lu w31_1 (HKAS 126514, holotype), ex-type culture CGMCC 3.23930 = UESTCC 22.0126; *ibid*., on dead culms of *Miscanthus sinensis* (Poaceae), ZH Lu Sarah57 (HUEST 22.0141), living culture UESTCC 22.0140; Chongzhou City, Baiyungou, 30°47′35″ N, 103°23′49″ E, elevation 990 m, on dead culms of *Imperata cylindrica* (Poaceae), 27 September 2021, ZH Lu Lu64 (HUEST 22.0142), living culture UESTCC 22.0141; Ngawa Tibetan and Qiang Autonomous Prefecture, Wenchuan County, Xiqiang Valley, 31°29′27″ N, 103°37′1″ E, elevation 1500 m, on dead culms of *Phragmites australis* (Poaceae), 20 October 2021, ZH Lu w168_1 (HUEST 22.0143), living culture UESTCC 22.0142; *ibid.* on dead culms of *Neyraudia reynaudiana* (Poaceae), ZH Lu w234_3 (HUEST 22.0144), living culture UESTCC 22.0143.

*Culture characteristics*: Colony on PDA reaching 37 mm diam after 16 days in an incubator under dark conditions at 20 °C, circular, cottony, hairy at the margin, white; reverse: white at the margin and pale yellow at the middle.

*Notes*: The phylogenetic tree shows the isolates UESTCC 22.0126, UESTCC 22.0140, UESTCC 22.0141, UESTCC 22.0142, and UESTCC 22.0143 form a clade sister to the isolates *P. chimonanthi* including the ex-type KUMCC 20-0266 (100% ML, 1.00 PP; Figure 1). *Periconia chimonanthi* was introduced based on the collection from decaying branches of *Chimonanthi praecox* (Calycanthaceae) in China [8]. Our collections have similar morphological characteristics in the shape of conidiophores and conidia with the *P. chimonanthi* on natural substrate. However, *P. chengduensis* differs from *P. chimonanthi* in having shorter conidiophores (240–370 μm vs. 410–635 μm) [8]. Furthermore, the culture of *P. chimonanthi* is black on the reverse side of PDA media; however, *P. chengduensis* (UESTCC 22.0126) is generally pale yellow on the reverse [8]. Thus, considering the difference in morphological characteristics and phylogenetic analysis, we describe the isolates (UESTCC 22.0126, UESTCC 22.0140, UESTCC 22.0141, UESTCC 22.0142, UESTCC 22.0143) as a new species.

***Periconiachimonanthi*** E.F. Yang, H.B. Jiang, and Phookamsak, in Yang et al. Journal of Fungi 8(3): 243 (2022) Figure 4.

*MycoBank*: MB 559497

*Saprobic* on dead leaves of Poaceae. **Asexual morph**: *Colonies* on the natural substrate numerous, effuse, dark brown to black, hairy. *Conidiophores* 200–345 μm long ($\bar{x}$ = 275, *n* = 15), 8–13 μm wide ($\bar{x}$ = 11 μm, *n* = 15), macronematous, mononematous, straight or slightly flexuous, unbranched, solitary, dark brown to black, septate, smooth to minutely verruculose, thick-walled. *Conidiogenous cells* polyblastic, pale brown to brown, terminal, integrated, subglobose, smooth to verruculose. *Conidia* 5.5–9 × 5.5–8.5 μm ($\bar{x}$ = 7.5 × 7.0 μm, *n* = 40), solitary or catenate, oval to globose, brown, aseptate, echinulate or verruculose. **Sexual morph**: Undetermined.

*Materials examined*: China, Sichuan Province, Chengdu City, Guoxue Park, 30°44′36″ N, 103°55′8″ E, elevation 506 m, on dead leaves of *Arundo donax* (Poaceae), 16 September 2021, ZH Lu Lu2_2 (HUEST 22.0134), living culture UESTCC 22.0133; *ibid*., on dead leaves of *Imperata cylindrica* (Poaceae), ZH Lu Lu8 (HUEST 22.0145), living culture UESTCC 22.0144.

*Culture characteristics*: Colony on PDA reaching 55 mm diam after 19 days in an incubator under dark conditions at 20 °C, oval almost circular, cottony, hairy at the margin, white at the margin, and gray-green at the middle; reverse: white at the margin and mud yellow to black toward the center.

*Notes*: The phylogenetic tree showed that our two isolates (UESTCC 22.0133, UESTCC 22.0144) clustered with other *P. chimonanthi* isolates, including type isolate (KUMCC 20-0266), that were introduced from decaying branches of *Chimonanthi praecox* (Calycanthaceae) in China [8]. Our two isolates display similar and overlapping morphological characteristics with the holotype of *P. chimonanthi* [8]. We identified our two collections (UESTCC 22.0133, UESTCC 22.0144) as *P. chimonanthi*, and this is the first report of *P. chimonanthi* isolated from *Arundo donax* and *Imperata cylindrica*.

***Periconia cookei*** E.W. Mason and M.B. Ellis, Mycol. Pap. 56: 72 (1953) Figure 5.

*MycoBank*: MB 302477

*Saprobic* on dead culms of Poaceae. **Asexual morph**: *Colonies* on the natural substrate numerous, effuse, brown to black, hairy. *Conidiophores* 310–515 μm long ($\bar{x}$ = 365, *n* = 15), 8.5–14.5 μm wide ($\bar{x}$ = 11 μm, *n* = 15), macronematous, mononematous, straight or slightly flexuous, branched, solitary, rarely 1–2 together on stroma, dark brown to black, 3–7-septate, smooth to minutely verruculose, thick-walled. *Conidiogenous cells* polyblastic, yellowish-brown to brown, terminal, integrated, oval to subglobose, smooth to verruculose. *Conidia* 5–8 × 5–7 μm ($\bar{x}$ = 6.5 × 6 μm, *n* = 40), solitary or catenate, oval to globose, brown to dark brown, aseptate, echinulate or verruculose. **Sexual morph**: Undetermined.

*Material examined*: China, Sichuan Province, Chongzhou City, Baiyungou, 30°47′35″ N, 103°23′49″ E, elevation 990 m, on dead culms of *Digitaria sanguinalis* (Poaceae), 27 September 2021, Z.H. Lu, Lu49_1 (HUEST 22.0135), living culture UESTCC 22.0134.

*Culture characteristics*: Colony on PDA reaching 39 mm diam after 24 days in an incubator under dark conditions at 20 °C, white, irregular circular, cottony, hairy at the margin; in reverse white at the margin and dark green to black at the middle.

*Notes*: *Periconia cookei* was introduced by Mason and Ellis [33] based on the morphology, characterized by conidiophores that are unbranched, septate, pale brown to dark brown, polyblastic and 13–16 μm diameter, catenate, verrucose, brown, mostly spherical conidia. The phylogenetic tree showed that our isolate UESTCC 22.0134 from the dead culms of *Digitaria sanguinalis* clustered with the isolate of *P. cookie* MFLUCC 17–1679 [54]. Thus, we identified the isolate UESTCC 22.0134 as a *P. cookie*. This is the first report of *P. cookie* occurring on *Digitaria sanguinalis* in Sichuan Province, China.

**Figure 6:**
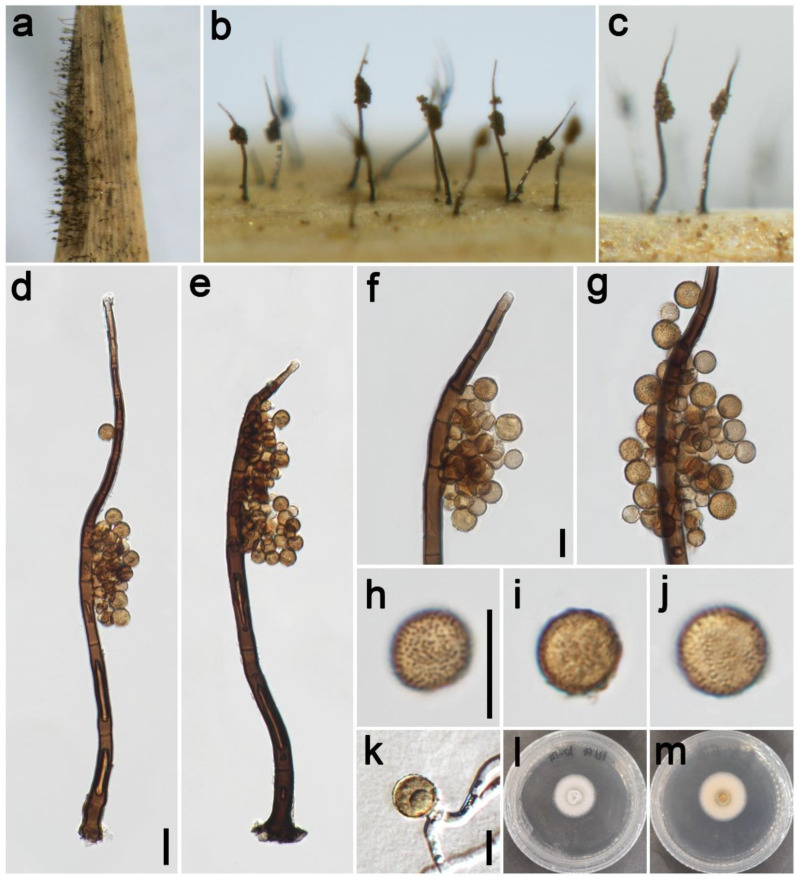
*Periconia cynodontis* (HKAS 126515, holotype). (**a**–**c**) Colonies on the natural substrate; (**d**,**e**) conidiophores; (**f**,**g**) conidiophores bearing conidia laterally; (**h**–**j**) conidia; (**k**) germinating conidium; (**l**,**m**) colony on PDA from above and below. Scale bars: (**d**) = 20 μm, (**f**,**h**,**k**) = 10 μm. Scale bar of (**d**) applies to (**e**). Scale bar of (**f**) applies to (**g**). Scale bar of (**h**) applies to (**i**,**j**).

***Periconia cynodontis*** Z.H. Lu, P.W. Su, and Maharachch., sp. nov. Figure 6.

*MycoBank*: MB 847465

*Etymology*: Name reflects the host genus, *Cynodon*, from which the fungus was collected.

*Saprobic* on dead leaves of Poaceae. **Asexual morph:** *Colonies* on natural substrate effuse, brown to dark brown, hairy. *Mycelium* mostly immersed, septate, smooth, brown. *Conidiophores* 240–335 μm long ($\bar{x}$ = 310, *n* = 15), 6.5–10.5 μm wide ($\bar{x}$ = 8.5 μm, *n* = 15), macronematous, mononematous, with setiform apices, erect, often curved, solitary, rarely 1–2 together on stroma, unbranched, 6–10-septate, brown to dark brown toward the base, thick-walled, smooth. *Conidiogenous cells* monoblastic or polyblastic, discrete, directly formed in the middle part of the conidiophores, unilateral, spherical to oval, pale brown to brown, smooth. *Conidia* 7.5–12.5 × 7–12 μm ($\bar{x}$ = 10.5 × 10 μm, *n* = 40), solitary or catenate, globose, brown to dark brown, aseptate, echinulate or verruculose. **Sexual morph:** Undetermined.

*Material examined*: China, Sichuan Province, Chengdu City, Guoxue Park, 30°44′36″ N, 103°55′8″ E, elevation 506 m, on dead leaves of *Cynodon dactylon* (Poaceae), 16 September 2021, ZH Lu Lu4 (HKAS 126515, holotype), ex-type culture CGMCC 3.23927 = UESTCC 22.0127.

*Culture characteristics*: Colony on PDA reaching 33 mm diam after one week in an incubator under dark conditions at 20 °C, white, circular, cottony, hairy at the margin; in reverse creamy white at the margin and pale yellow at the middle.

*Notes*: The phylogenetic tree shows that the isolate *Periconia cynodontis* UESTCC 22.0127 formed a distinct clade within *Periconia*. *Periconia cynodontis* resembles *P*. *lateralis* in having curved conidiophores, conidiogenous cells directly formed in the middle part of the conidiophores, and solitary or catenate, globose, brown to dark brown conidia [55,56]. However, *P. cynodontis* differs from *P. lateralis* by having smaller conidia (7.5–12.5 μm vs. 10.5–15 µm) [55]. *Periconia cynodontis* differs from *P*. *penniseti* and *P. neobrittanica* by conidiogenous cells that are directly formed in the middle part of the conidiophores [57]. Thus, considering the difference in morphological characteristics and phylogenetic analysis, we describe the isolate UESTCC 22.0127 as *Periconia cynodontis* sp. nov.

**Figure 7:**
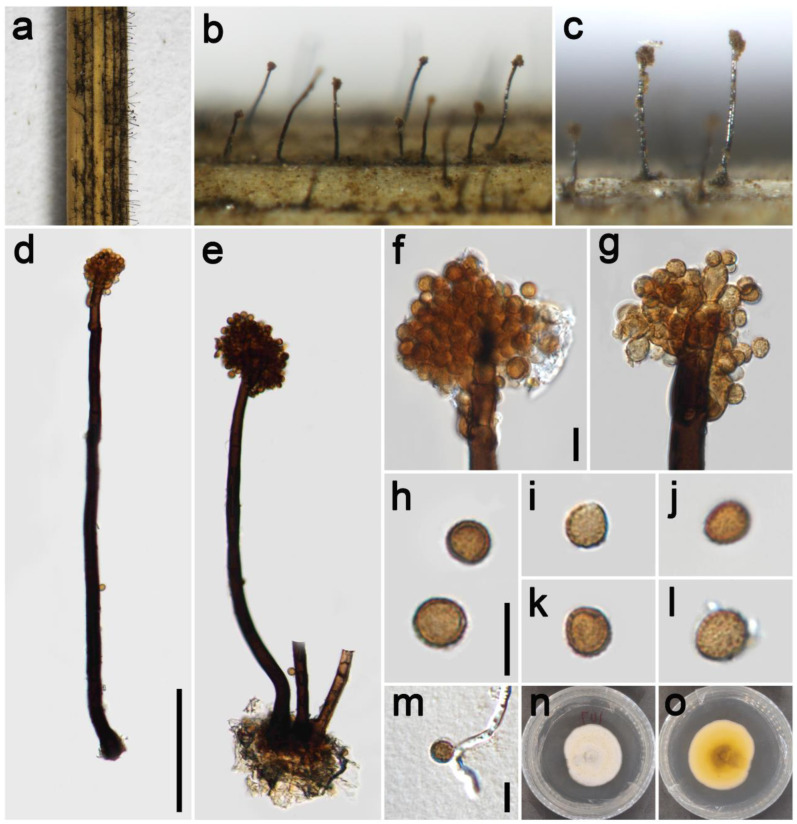
*Periconia festucae* (HKAS 126516, holotype). (**a**–**c**) Colonies on the natural substrate; (**d**,**e**) conidiophores with spherical conidial heads; (**f**,**g**) apical branch conidiophores with conidial head; (**h**–**l**) conidia; (**m**) germinating conidium; (**n**,**o**) colony on PDA from above and below. Scale bars: (**d**) = 100 μm, (**f**,**h**,**m**) = 10 μm. Scale bar of (**d**) applies to (**e**). Scale bar of (**f**) applies to (**g**). Scale bar of (**h**) applies to (**i**–**l**).

***Periconia festucae*** Z.H. Lu, P.W. Su, and Maharachch., sp. nov. Figure 7. 

*MycoBank*: MB 847466

*Etymology*: Name after the host genus from which the fungus was collected, *Festuca*.

*Saprobic* on dead culms of of Poaceae. **Asexual morph:** *Colonies* on the natural substrate numerous, effuse, dark brown to black, hairy. *Conidiophores* 225–495 μm long ($\bar{x}$ = 310, *n* = 15), 10.5–20 μm wide ($\bar{x}$ = 13 μm, *n* = 15), macronematous, mononematous, straight or slightly flexuous, solitary or gregarious (1–3 together on substrate), brown to dark brown, 4–8-septate, smooth to minutely verruculose, thick-walled. *Conidiogenous cells* polyblastic, pale brown to brown, terminal, integrated or discrete, subglobose to globose, smooth to verruculose. *Conidia* 5–8 × 4.8–8 μm ($\bar{x}$ = 6.5 × 6.4 μm, *n* = 40), solitary, oval to globose, light brown to dark brown, aseptate, verruculose. **Sexual morph:** Undetermined.

*Material examined*: China, Sichuan Province, Chengdu City, Guoxue Park, 30°44′36″ N, 103°55′8″ E, elevation 506 m, on dead culms of *Festuca elata* (Poaceae), 16 September 2021, ZH Lu Lu7 (HKAS 126516, holotype), ex-type culture CGMCC 3.23929 = UESTCC 22.0128.

*Culture characteristics*: Colony on PDA reaching 47 mm diam after 19 days in an incubator under dark conditions at 20 °C, oval to almost circular, cottony, hairy and white at the margin, and pale yellow to white at the middle; in reverse yellow at the margin and mud yellow at the middle.

*Notes*: The isolate UESTCC 22.0128 is grouped in a well-supported clade and appears to be phylogenetically distinct (98% ML, 0.95 PP; Figure 1) from other sister species. Our collection HKAS 126516 shares similar morphological characteristics in the shape and color of conidiophores and conidia with the *P*. *cookie* and *P*. *verrucosa* on the natural substrate [54]. However, it differs from *P*. *cookie* and *P*. *verrucosa* by having significantly wider conidiophores (10.5–20 μm vs. 8.5–14.5 μm and 10.5–15 μm) [34]. The culture of *P. cookie* is dark green to black on the reverse side of PDA media. However, *P*. *festucae* is yellow on the reverse. *Periconia festucae* and *P*. *verrucosa* differ in the number of septa on conidiophores (4–8-septate vs. 2–4-septate). Considering the significant differences in morphology and molecular data, we introduce the isolate UESTCC 22.0128 as a new species.

**Figure 8:**
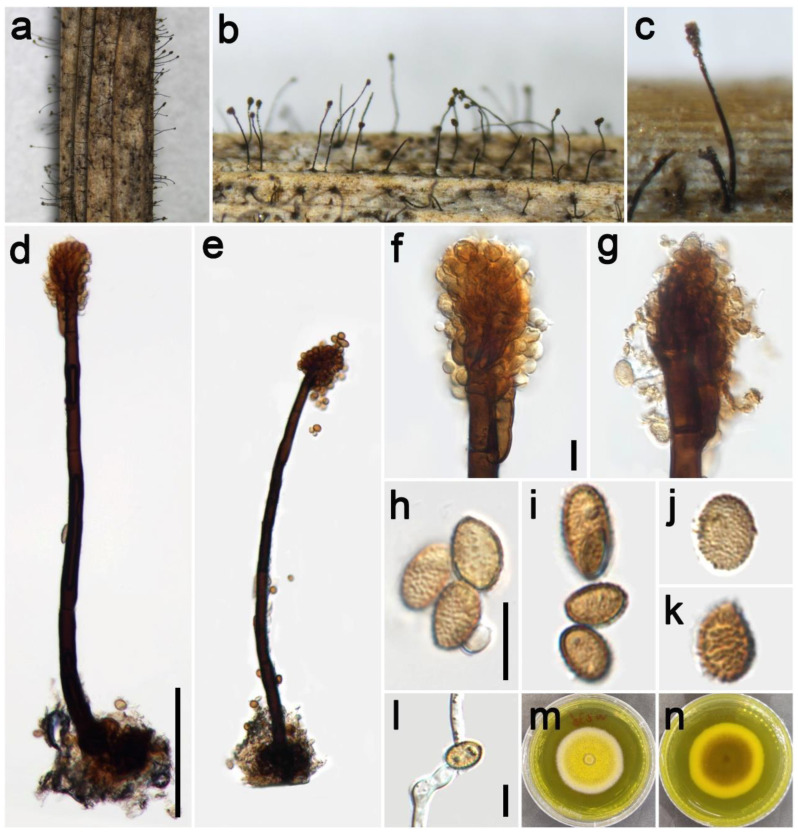
*Periconia imperatae* (HKAS 126517, holotype). (**a**–**c**) Colonies on the natural substrate; (**d**,**e**) conidiophores with spherical conidial heads; (**f**,**g**) apically branch conidiophores with conidial head; (**h**–**k**) conidia; (**l**) germinating conidium; (**m**,**n**) colony on PDA from above and below. Scale bars: (**d**) = 100 μm, (**f**,**h**,**l**) = 10 μm. Scale bar of (**d**) applies to (**e**). Scale bar of (**f**) applies to (**g**). Scale bar of (**h**) applies to (**i**–**k**).

***Periconia imperatae*** Z.H. Lu, P.W. Su, and Maharachch., sp. nov. Figure 8. 

*MycoBank*: MB 847467

*Etymology*: Name after the host genus from which the fungus was isolated, *Imperata*.

Saprobic dead leaves and culms of Poaceae. **Asexual morph:** *Colonies* on the natural substrate numerous, effuse, dark brown to black, floccose. *Conidiophores* 280–520 μm long ($\bar{x}$= 378, *n* = 15), 10–16.5 μm wide ($\bar{x}$ = 12.5 μm, *n* = 15), macronematous, mononematous, straight or slightly flexuous, branched, solitary, rarely 1–2 together on stroma, brown to dark brown, 3–7-septate, smooth to minutely verruculose, thick-walled. *Conidiogenous cells* polyblastic, pale brown to brown, terminal, integrated or discrete, oval to subglobose, smooth to verruculose. *Conidia* 8.5–13.5 × 5.5–8.5 μm ($\bar{x}$ = 10.5 × 7 μm, *n* = 40), solitary, ovoid, initially faint yellow or pale brown, becoming brown to dark brown at maturity, aseptate, verruculose. **Sexual morph:** Undetermined.

*Materials examined*: China, Sichuan Province, Ngawa Tibetan and Qiang Autonomous Prefecture, Wenchuan County, Xiqiang Valley, 31°29′27″ N, 103°37′1″ E, elevation 1500 m, on dead leaves and culms of *Imperata cylindrica* (Poaceae), 20 October 2021, ZH Lu w236 (HKAS 126517, holotype), ex-type culture CGMCC 3.23931 = UESTCC 22.0129; *ibid*., w230_1 (HUEST 22.0146), living culture UESTCC 22.0145; *ibid*., on dead culms of *Neyraudia reynaudiana* (Poaceae), ZH Lu w234_5 (HUEST 22.0147), living culture UESTCC 22.0146.

*Culture characteristics*: Colony on PDA reaching 58 mm diam after 11 days in an incubator under dark conditions at 20 °C, circular, cottony, hairy and white at the margin and yellow toward the middle; yellow at the margin and yellow to brown at the middle in reverse; producing yellow pigments on PDA. 

*Notes*: The phylogenetic tree shows that our three collections (UESTCC 22.0129, UESTCC 22.0145, UESTCC 22.01456) formed a distinct lineage within *Periconia* as the sister to the *P. submersa* (99% ML, 1.00 PP; Figure 1). *Periconia imperatae* resembles *P. submersa* in having ovoid conidia. However, it differs from *P. submersa* by having significantly larger conidia (8.5–13.5 × 5.5–8.8 μm vs. 6.5–9.5 × 4.5–5.5 μm) [12]. Furthermore, *P. imperatae* produced yellow pigment on PDA. Thus, considering the difference in morphology and phylogeny, we describe the isolate UESTCC 22.0129 as a new species.

**Figure 9:**
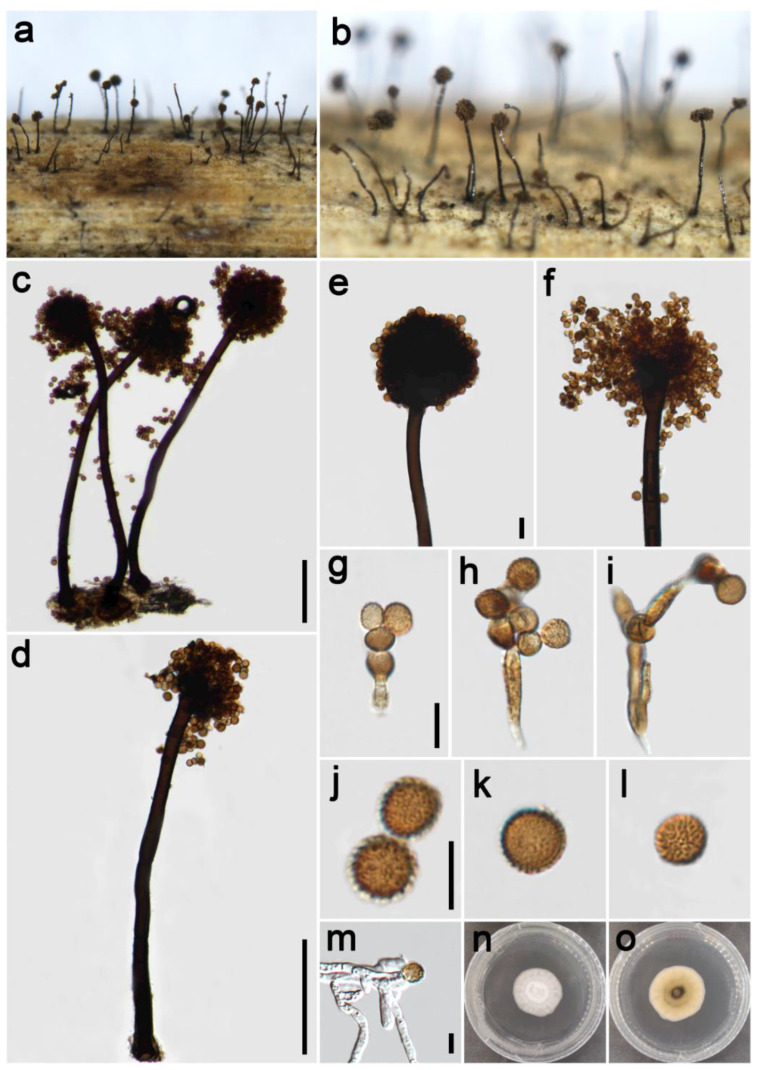
*Periconia penniseti* (HKAS 126518, holotype). (**a**,**b**) Colonies on the natural substrate; (**c**,**d**) conidiophores with spherical conidial heads; (**e**,**f**) conidial heads bearing conidiogenous cells and conidia; (**g**–**i**) conidiogenous cells bearing conidia in short chains; (**j**–**l**) conidia; (**m**) germinating conidium; (**n**,**o**) colony on PDA from above and below. Scale bars: (**c**,**d**) = 100 μm, (**e**,**g**,**j**,**m**) = 10 μm. Scale bar of (**e**) applies to (**f**). Scale bar of (**g**) applies to (**h**,**i**). Scale bar of (**j**) applies to (**k**,**l**).

***Periconia penniseti*** Z.H. Lu, P.W. Su, and Maharachch., sp. nov. Figure 9.

*MycoBank*: MB 847468

*Etymology*: Name reflects the host genus, *Pennisetum*, from which the fungus was collected.

*Saprobic* on dead culms of Poaceae. **Asexual morph:** *Colonies* on the natural substrate numerous, effuse, dark brown to black, hairy. *Conidiophores* 285–540 μm long ($\bar{x}$ = 370, *n* = 15), 11–17 μm wide ($\bar{x}$ = 14.5 μm, *n* = 15), macronematous, mononematous, straight to flexuous, unbranched, solitary or gregarious (1–3 together on substrate), dark brown to black, 2–5-septate, smooth to minutely verruculose, thick-walled, forming spherical conidial heads at apex. *Conidiogenous cells* polyblastic, pale brown to brown, occurring in an apical chain on primary or directly on conidiophore, integrated or discrete, smooth to verruculose. *Conidia* 6–11.5 × 6–11 μm ($\bar{x}$ = 9 × 8.5 μm, *n* = 40), catenate, globose, brown to dark brown, aseptate, verruculose. **Sexual morph:** Undetermined.

*Material examined*: China, Sichuan Province, Chengdu City, Guoxue Park, 30°44′36″ N, 103°55′8″ E, elevation 506 m, on dead culms of *Pennisetum* sp. (Poaceae), 16 September 2021, ZH Lu Lu5 (HKAS 126518, holotype), ex-type culture CGMCC 3.23928 = UESTCC 22.0130.

*Culture characteristics*: Colony on PDA 23 mm diam after 2 weeks in an incubator under dark conditions at 20 °C, irregular circular, cottony, hairy at the margin, colonies from above white grayish to creamy white; in reverse, white at the margin and pale yellow to khaki at the middle.

*Notes*: The phylogenetic tree shows that the isolate UESTCC 22.0130 clustered with the ex-type strain of *P. neobrittanica* (CBS 146062), which was isolated from leaves of *Melaleuca styphelioides* (Myrtaceae) in California, USA [57]. *Periconia penniseti* can be distinguished from *P. neobrittanica* in having longer conidiophores (285–540 μm vs. 100–300 μm) and that are more septate (2–5 vs. 0–1) [57]. *Periconia penniseti* differs from *P. cynodontis* in the position of conidiogenous cells. In *P. penniseti,* the conidiogenous cells arise from the apical part of the conidiophores, while in *P. cynodontis,* the conidiogenous cells arise from the middle part of the conidiophores. Thus, considering the difference in morphological characteristics and phylogenetic analysis, we describe the isolate UESTCC 22.0130 as a new species.

***Periconia pseudobyssoides*** Markovsk. and A. Kačergius, Mycol. Progr. 13(2): 293 (2014) Figure 10.

*MycoBank*: MB 804763

*Saprobic* on dead culms of Poaceae. **Asexual morph:** *Colonies* on natural substrate numerous, effuse, brown to dark brown, hairy. *Conidiophores* 300–565 μm long ($\bar{x}$ = 445, *n* = 15), 9.5–14.5 μm wide ($\bar{x}$ = 11.5 μm, *n* = 15), macronematous, mononematous, straight or slightly flexuous, solitary, pale brown to brown, 4–7-septate, smooth to minutely verruculose, thick-walled. *Conidiogenous cells* polyblastic, pale brown to brown, terminal, integrated, subglobose, smooth to verruculose. *Conidia* 10.5–17 × 10.5–16.5 μm ($\bar{x}$ = 14 × 13.5 μm, *n* = 40), solitary, globose, yellowish-brown to golden-brown, aseptate, echinulate, or verruculose. **Sexual morph:** Undetermined.

*Materials examined*: China, Sichuan Province, Chongzhou City, Baiyungou, 30°47′35″ N, 103°23′49″ E, elevation 990 m, on dead leaves of *Digitaria sanguinalis* (Poaceae), 27 September 2021, ZH Lu Lu39 (HUEST 22.0136), living culture UESTCC 22.0135; *ibid*., Lu96 (HUEST 22.0148), living culture UESTCC 22.0147.

*Culture characteristics*: Colony on PDA reaching 46 mm diam after 10 days in an incubator under dark conditions at 20 °C, irregular circular, cottony, hairy at the margin, white; reverse: pale yellow at the margin and brown to black at the middle.

*Notes*: The phylogenetic tree showed that our two isolates (UESTCC 22.0135, UESTCC 22.0147) clustered with other *P. pseudobyssoides* isolates. Morphologically, our two collections are similar to the description of *P. pseudobyssoides*. Thus, we identified our collections as *P. pseudobyssoides*. This is the first record of *Periconia* species occurring on *Digitaria sanguinalis*.

**Figure 11:**
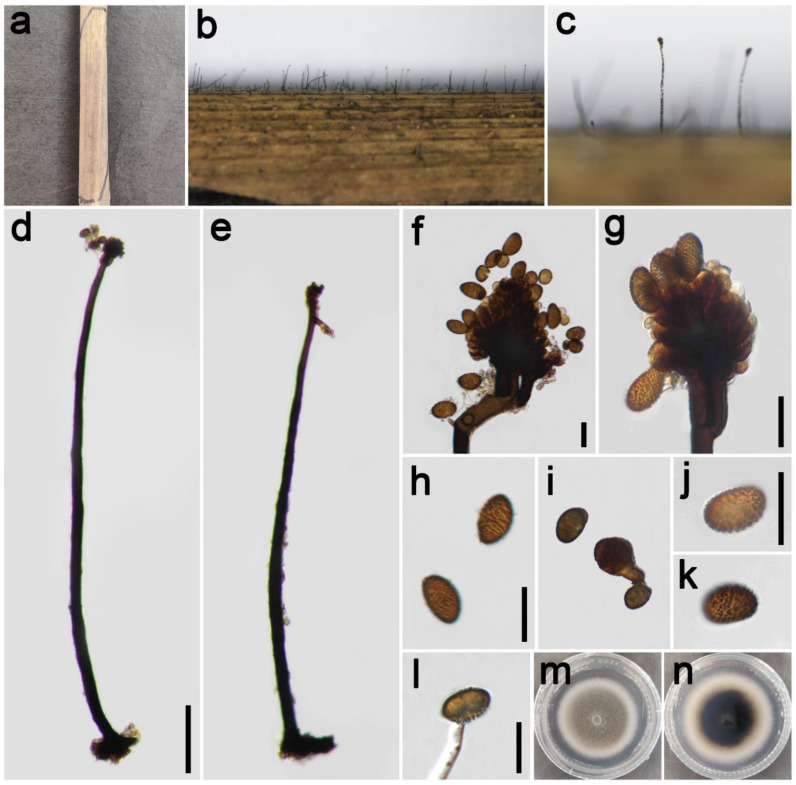
*Periconia spodiopogonis* (HKAS 126519, holotype). (**a**–**c**) Colonies on the natural substrate; (**d**,**e**) conidiophores; (**f**,**g**) apically branch conidiophores with conidial head; (**h**–**k**) conidia; (**l**) germinating conidium; (**m**,**n**) colony on PDA from above and below. Scale bars: (**d**) = 100 μm, (**f**,**g**,**h**,**j**,**l**) = 20 μm. Scale bar of (**d**) applies to (**e**). Scale bar of (**h**) applies to (**i**). Scale bar of (**j**) applies to (**k**).

***Periconia spodiopogonis*** Y.P. Chen, Z.H. Lu, P.W. Su, and Maharachch., sp. nov. Figure 11. 

*MycoBank*: MB 847469

*Etymology*: Name reflects the host genus, *Spodiopogon*, from which the fungus was collected.

*Saprobic* on dead culms of *Spodiopogon ludingensis*. **Asexual morph:** *Colonies* on the natural substrate numerous, effuse, dark brown to black, hairy. *Conidiophores* 460–720 μm long ($\bar{x}$ = 595, *n* = 15), 9–18 μm wide ($\bar{x}$ = 15 μm, *n* = 15), macronematous, mononematous, straight or slightly flexuous, branched, solitary, dark brown to black, slightly paler toward the apex, septate, smooth to minutely verruculose, thick-walled. *Conidiogenous cells* polyblastic, brown, terminal, integrated or discrete, oval to subglobose, smooth to verruculose. *Conidia* 14.5–24.5 × 9.5–15.5 μm ($\bar{x}$ = 20 × 12.5 μm, *n* = 40), solitary, ellipsoidal to cylindrical, brown to dark brown, aseptate, verruculose. **Sexual morph:** Undetermined.

*Material examined*: China, Sichuan Province, Pengzhou City, Huilonggou, 31°18′11″ N, 103°45′35″ E, elevation 1400 m, on dead culms of *Spodiopogon ludingensis* (Poaceae), 28 July 2021, YP Chen 2021072818 (HKAS 126519, holotype), ex-type culture CGMCC 3.23932 = UESTCC 22.0131.

*Culture characteristics*: Colony on PDA reaching 68 mm diam after 10 days in an incubator under dark conditions at 20 °C, circular, cottony, hairy, and pale gray at the margin and gray to pale brown at the center; gray at the margin and dark brown to black at the middle in reverse.

*Notes*: The phylogenetic tree shows that the isolate UESTCC 22.0131 clustered with the ex-type of *P. submersa* (MFLUCC 16-1098), which was introduced from submerged decaying wood in the Nujiang River, Yunnan Province, China [12]. *Periconia spodiopogonis* shares similar morphological characteristics, such as the shape of conidiophores and conidia, with the holotype of the *P. submersa* (HKAS 92738) [12]. However, it differs from *P. submersa* by having significantly longer conidiophores (460–720 μm vs. 485.5–529.5 μm) and larger conidia (14.5–24.5 × 9.5–15.5 μm vs. 6.5–9.5 × 4.5–5.5 μm) [12]. The BLASTn analysis of *P. submersa* (MFLUCC 16-1098) and *P. spodiopogonis* (CGMCC 3.23932) shows 96% identity (484/504, 4 gaps) using ITS. Considering the significant differences in morphology and sequence data, we introduce the isolate UESTCC 22.0131 as a *Periconia spodiopogonis* sp. nov.

***Periconia verrucosa*** Phukhams., Ertz, Gerstmans, and K.D. Hyde, in Phukhamsakda et al. Fungal Diversity, 102(1): 71 (2020) Figure 12.

*MycoBank*: MB 557143

*Saprobic* on dead leaves of Poaceae. **Asexual morph:** *Colonies* on the natural substrate numerous, effuse, dark brown, hairy. *Conidiophores* 353–545 μm long ($\;\bar{x}\;$= 410, *n* = 15), 10.5–15 μm wide ($\bar{x}$ = 12.5 μm, *n* = 15), macronematous, mononematous, straight or flexuous, branched, solitary, dark brown to black, 2–4-septate, smooth to minutely verruculose, thick-walled. *Conidiogenous cells* polyblastic, yellow to pale brown, terminal, integrated, subglobose, smooth to verruculose. *Conidia* 4.5–8 × 4.5–7.5 μm ($\bar{x}$ = 6 × 5.5 μm, *n* = 40), solitary or catenate, oval to globose, pale brown to dark brown, aseptate, echinulate or verruculose. **Sexual morph:** Undetermined.

*Materials examined:* China, Sichuan Province, Chongzhou City, Baiyungou, 30°47′35″ N, 103°23′49″ E, elevation 990 m, on dead leaves of *Stipa tenuissima* (Poaceae), 27 September 2021, ZH Lu Lu40_1 (HUEST 22.0137), living culture UESTCC 22.0136; *ibid*., on dead culms of *Phyllostachys nigra* (Poaceae), Lu53_1 (HUEST 22.0149), living culture UESTCC 22.0148; *ibid*., Lu98_2 (HUEST 22.0150), living culture UESTCC 22.0149; Ngawa Tibetan and Qiang Autonomous Prefecture, Wenchuan County, Xiqiang Valley, 31°29′27″ N, 103°37′1″ E, elevation 1500 m, on dead culms of *Pennisetum polystachion* (Poaceae), 20 October 2021, ZH Lu w232_2 (HUEST 22.0151), living culture UESTCC 22.0150; *ibid*., on dead culms of *Neyraudia reynaudiana* (Poaceae), w234_4 (HUEST 22.0152), living culture UESTCC 22.0151.

*Culture characteristics:* Colony on PDA reaching 37 mm diam after 19 days in an incubator under dark conditions at 20 °C, white, circular, cottony, hairy at the margin; in reverse pale yellow at the margin and yellow toward the center.

*Notes:* The phylogenetic tree showed our five isolates (UESTCC 22.0136, UESTCC 22.0148, UESTCC 22.0149, UESTCC 22.0150, UESTCC 22.0151) clustered with the existing *P. verrucosa* collections including the type (MFLUCC 17–2158). The type species of *P. verrucosa* was isolated from decaying stems of *Clematis viticella* in Belgium [34]. Morphologically, there are no significant differences between our collections and the type species of *P. verrucosa*. Therefore, our five collections are identified as *P. verrucosa*, and this is the first report of *P. verrucosa* from terrestrial habitats in China.

## 4. Discussion

Species of *Periconia* have been reported from a large number of host plants, including graminaceous plants [4,8,55]. For example, previously, *P. hispidula* and *P. thysanolaenae* were recorded from graminaceous plants in China [8,58]. It is known that this genus produces a number of bioactive secondary metabolites, including terpenes, polyketides, aromatic compounds, and carbohydrate derivatives [59]. *Periconia* sp. isolated from *Torreya grandifolia* produced taxol, known as an anticancer compound [60]. Similarly, compounds 2,4-dihydroxy-6-[(1′E,3′E)-penta-1′,3′-dienyl]-benzaldehyde isolated from *P. atropurpurea* had vigorous antifungal activity against *Cladosporium sphaerospermum* and *C. cladosporioides* [19]. Therefore, the genus *Periconia* has excellent potential for natural product exploration and the development of pharmacological agents. Thus, future genetic and secondary metabolites studies on this chemically highly diverse genus may lead to the discovery of novel biochemical properties unique to the *Periconia* group.

Sichuan Province along the Yangtze River is a biodiversity hot spot [61,62]. In Sichuan Province, we regularly conduct fungal diversity surveys. This survey collected samples from five sites; 26 *Periconia* strains were morphologically and phylogenetically characterized, which resulted in 11 species identified and six new taxa described. In the phylogenetic tree, *P. salina* was clustered within *P. byssoides*, but Yang et al. [8] showed that *P. byssoides* and *P. salina* were not conspecific by the pairwise homogeneity index (PHI) test. *Periconia* is an ancient and species-rich genus and among 130 morphologically accepted *Periconia* species [8]. However, only 43 species (including six new species in this study) have molecular data in GenBank. Even though many *Periconia* species only have ITS and LSU loci. As a highly diverse group, it is also difficult to distinguish species within *Periconia* using only ITS and LSU gene regions. Therefore, in future studies, more phylogenetic markers with high resolving power are essential to understand the species boundaries in this highly diverse genus. In addition, due to a lack of information and sequence data on many of the species, including the type of *Periconia*, epitype with living cultures are essential for further comprehensive studies to clarify the taxonomic status of the genus.

## Figures and Tables

**Figure 2:**
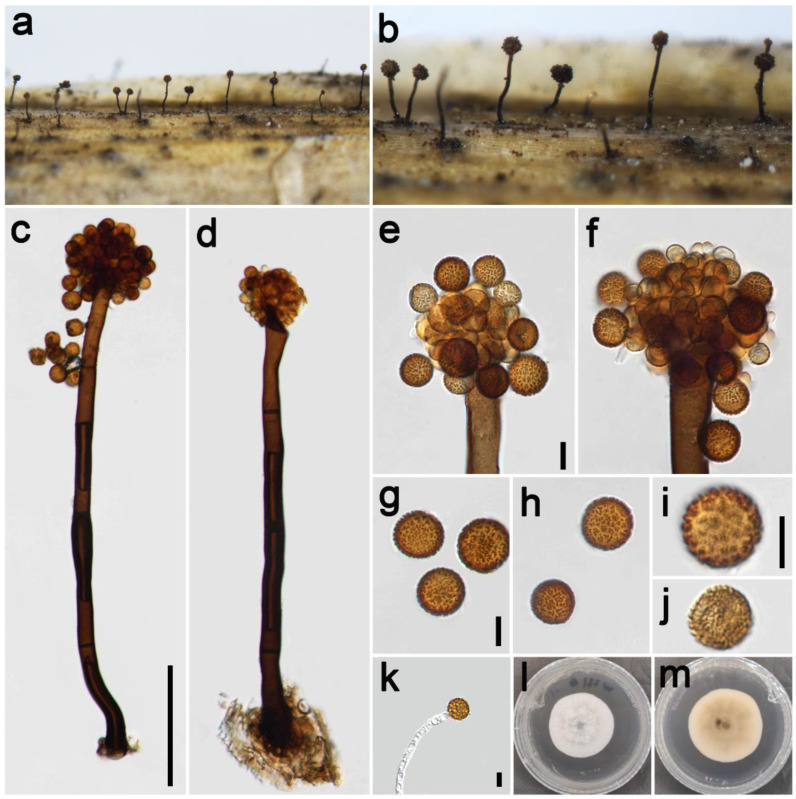
*Periconia byssoides* (HUEST 22.0133). (**a**,**b**) Colonies on the natural substrate; (**c**,**d**) conidiophores with spherical conidial heads; (**e**,**f**) conidial heads bearing conidiogenous cells and conidia; (**g**–**j**) conidia; (**k**) germinating conidium; (**l**,**m**) colony on PDA from above and below. Scale bars: (**c**) = 100 μm, (**e**,**g**,**k**) = 10 μm. Scale bar of (**c**) applies to (**d**). Scale bar of (**e**) applies to (**f**). Scale bar of (**g**) applies to (**h**). Scale bar of (**i**) applies to (**j**).

**Figure 4:**
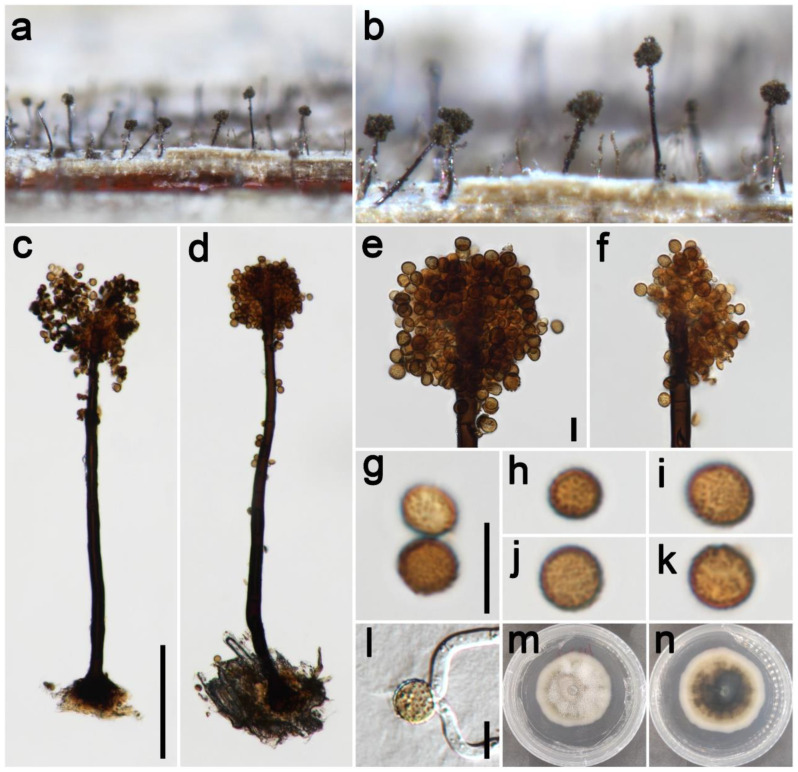
*Periconia chimonanthi* (HUEST 22.0134). (**a**,**b**) Colonies on the natural substrate; (**c**,**d**) conidiophores with spherical conidial heads; (**e**,**f**) conidial heads bearing conidiogenous cells and conidia; (**g**–**k**) conidia; (**l**) germinating conidium; (**m**,**n**) colony on PDA from above and below. Scale bars: (**c**) = 100 μm, (**e**,**g**,**i**) = 10 μm. Scale bar of (**c**) applies to (**d**). Scale bar of (**e**) applies to (**f**). Scale bar of (**g**) applies to (**h**–**k**).

**Figure 5:**
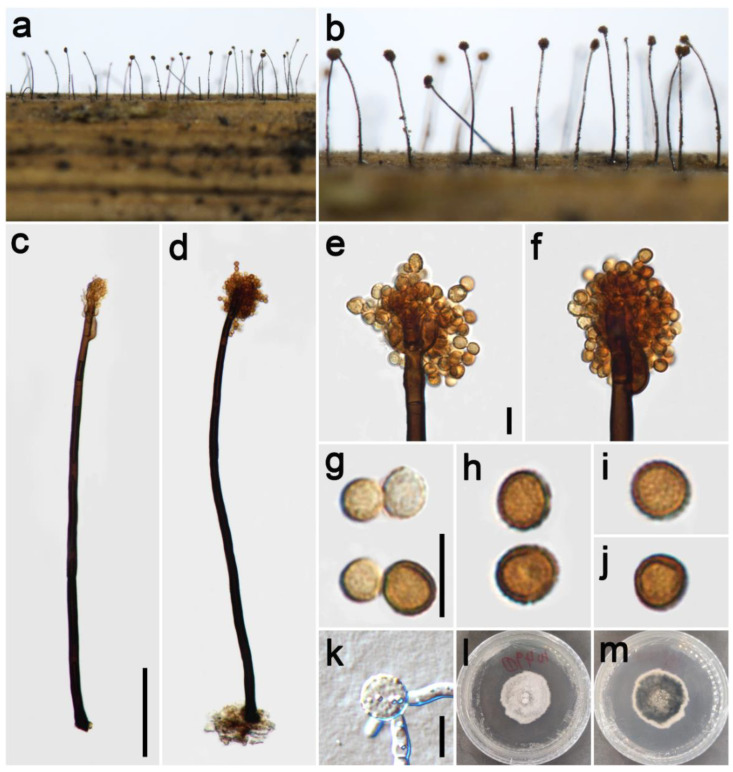
*Periconia cookei* (HUEST 22.0135). (**a**,**b**) Colonies on the natural substrate; (**c**,**d**) conidiophores with spherical conidial heads; (**e**,**f**) apically branch conidiophores with conidial head; (**g**–**j**) conidia; (**k**) germinating conidium; (**l**,**m**) colony on PDA from above and below. Scale bars: (**c**) = 100 μm, (**e**,**g**,**k**) = 10 μm. Scale bar of (**c**) applies to (**d**) Scale bar of (**e**) applies to (**f**). Scale bar of (**g**) applies to (**h**–**j**).

**Figure 10:**
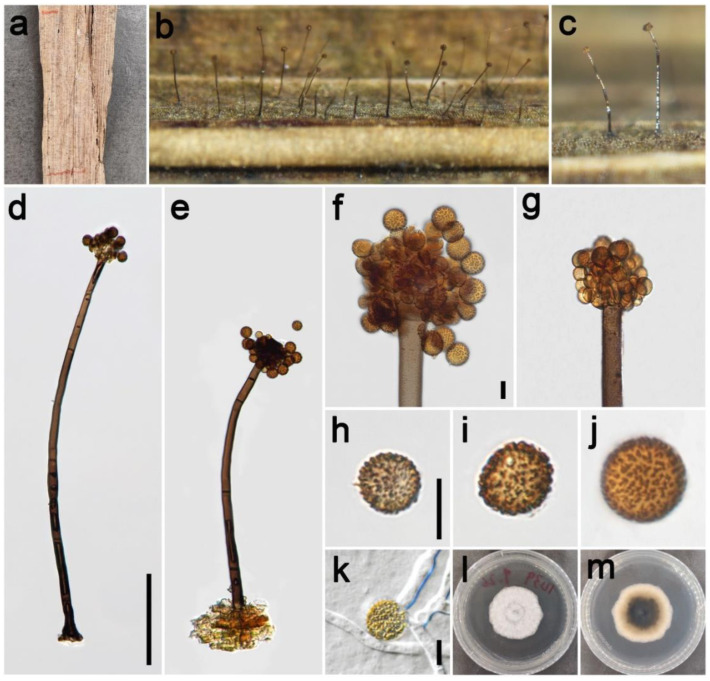
*Periconia pseudobyssoides* (HUEST 22.0136). (**a**–**c**) Colonies on the natural substrate; (**d**,**e**) conidiophores with spherical conidial heads; (**f**,**g**) conidial heads bearing conidiogenous cells and conidia; (**h**–**j**) conidia; (**k**) germinating conidium; (**l**,**m**) colony on PDA from above and below. Scale bars: (**d**) = 100 μm, (**f**,**h**,**k**) = 10 μm. Scale bar of (**d**) applies to (**e**). Scale bar of (**f**) applies to (**g**). Scale bar of (**h**) applies to (**i**,**j**).

**Figure 12:**
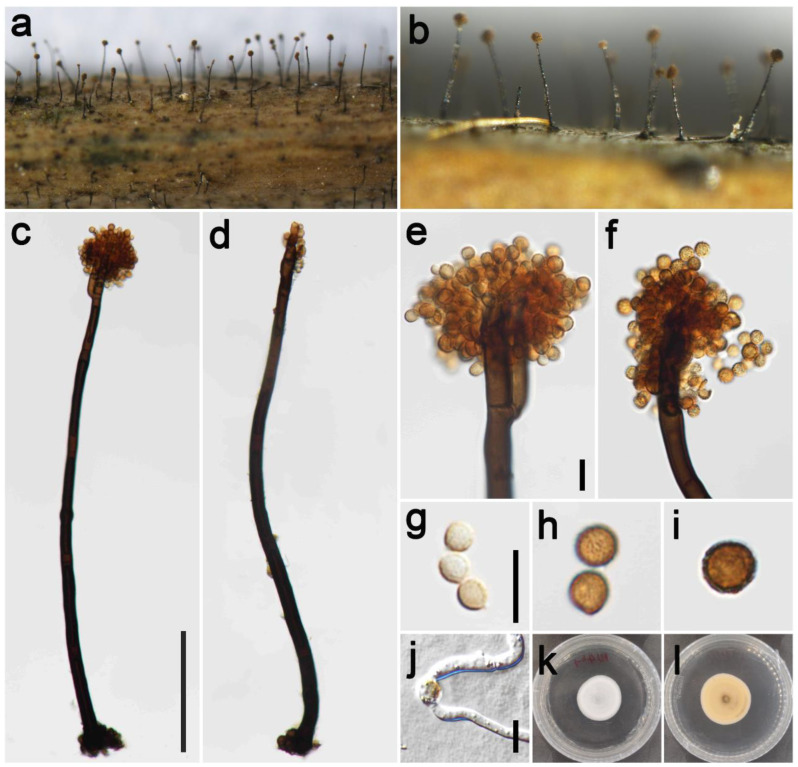
*Periconia verrucosa* (HUEST 22.0137). (**a**,**b**) Colonies on the natural substrate; (**c**,**d**) conidiophores with spherical conidial heads; (**e**,**f**) apically branch conidiophores with conidial head; (**g**–**i**) conidia; (**j**) germinating conidium; (**k**,**l**) colony on PDA from above and below. Scale bars: (**c**) = 100 μm, (**e**,**g**,**j**) = 10 μm. Scale bar of (**c**) applies to (**d**). Scale bar of (**e**) applies to (**f**). Scale bar of (**g**) applies to (**h**–**i**).

**Table 1:** Loci used in this study with the corresponding PCR primers and conditions.

Locus	PCR Primers	PCR Thermal Cycles	References
ITS	ITS9mun/ITS4_KYO1	(94 °C: 3 min) × 1 cycles (94 °C: 30 s, 56 °C: 30 s, 72 °C: 30 s) × 35 cycles (72 °C: 5 min) × 1 cycles	[39]
LSU	LR0R/LR5	(94 °C: 3 min) × 1 cycles (94 °C: 30 s, 56 °C: 30 s, 72 °C: 1 min) × 35 cycles (72 °C: 5 min) × 1 cycles	[40]
SSU	PNS1/NS41	(94 °C: 3 min) × 1 cycles (94 °C: 30 s, 56 °C: 30 s, 72 °C: 1 min) × 35 cycles (72 °C: 5 min) × 1 cycles	[41]
*TEF1*	EF1-983/EF1-2218R or TEF1LLErev	(94 °C: 3 min) × 1 cycles (94 °C: 30 s, 52 °C: 30 s, 72 °C: 1 min) × 35 cycles (72 °C: 5 min) × 1 cycles	[42,43]
*RPB2*	dRPB2-5f or RPB2-5F2/fRPB2-7cR	(94 °C: 3 min) × 1 cycles (94 °C: 30 s, 52 °C: 30 s, 72 °C: 1 min) × 35 cycles (72 °C: 5 min) × 1 cycles	[44,45]

**Table 2:** Species details and their GenBank accession numbers used in phylogenetic analyses.

Organism	Culture/Specimen No.	SSU	ITS	LSU	*TEF1*	*RPB2*
*Massarina cisti*	CBS 266.62	FJ795490	LC014568	AB807539	AB808514	FJ795464
*Periconia algeriana*	CBS 321.79	-	MH861212	MH872979	-	-
*P. alishanica*	KUMCC 19–0174 = NCYU19–0347	-	MW063167	MW063231	MW183792	-
***P. alishanica***	**MFLUCC 19–0145**	**-**	**MW063165**	**MW063229**	**MW183790**	**-**
*P. alishanica*	NCYUCC 19–0186	-	MW063166	MW063230	MW183791	-
***P. aquatica***	**MFLUCC 16–0912**	**-**	**KY794701**	**KY794705**	**KY814760**	**-**
***P. artemisiae***	**KUMCC 20–0265**	**MW448658**	**MW448657**	**MW448571**	**MW460898**	**-**
*P. atropurpurea*	CBS 381.55	-	MH857524	MH869061	-	-
***P. banksiae***	**CBS 129526 = CPC 17282**	**-**	**JF951147**	**NG_064279**	**-**	**-**
*P. byssoides*	MFLUCC 17–2292 = KUMCC 18–0272 = C292	MK347858	MK347751	MK347968	MK360069	MK434886
*P. byssoides*	MFLUCC 18–1548	MK347902	MK347794	MK348013	-	MK434863
*P. byssoides*	MFLUCC 18–1553 = C457	MK347914	MK347806	MK348025	MK360068	MK434858
*P. byssoides*	MFLUCC 19–0134 = NCYUCC 19–0166 = MFLU 18–2545	-	MW063164	MW063228	MW183789	-
***P. byssoides***	**MFLUCC 20–0172 = NCYUCC 19–0313**	**-**	**MW063162**	**MW063226**	**-**	**-**
*P. byssoides*	NCYUCC 19–0314 = NYCU 19–0040	-	MW063163	MW063227	-	-
*P. byssoides*	UESTCC 22.0132	OP956054	OP955985	OP956010	OP961451	OP961468
*P. byssoides*	UESTCC 22.0137	OP956036	OP955967	OP955992	OP961433	OP961458
*P. byssoides*	UESTCC 22.0138	OP956038	OP955969	OP955994	OP961435	OP961461
*P. byssoides*	UESTCC 22.0139	OP956055	OP955986	OP956011	OP961452	-
***P. caespitosa***	**LAMIC 110/16**	**-**	**MH051906**	**MH051907**	**-**	**-**
***P. chengduensis***	**CGMCC 3.23930 = UESTCC 22.0126**	**OP956056**	**OP955987**	**OP956012**	**OP961453**	**OP961469**
*P. * *chengduensis*	UESTCC 22.0140	OP956046	OP955977	OP956002	OP961443	OP961465
*P. * *chengduensis*	UESTCC 22.0141	OP956041	OP955972	OP955997	OP961438	-
*P. * *chengduensis*	UESTCC 22.0142	OP956047	OP955978	OP956003	OP961444	-
*P. * *chengduensis*	UESTCC 22.0143	OP956050	OP955981	OP956006	OP961447	OP961466
***P. chimonanthi***	**KUMCC 20–0266**	**MW448656**	**NR_176752**	**MW448572**	**MW460897**	**-**
*P. chimonanthi*	UESTCC 22.0133	OP956033	OP955964	OP955989	OP961430	OP961455
*P. chimonanthi*	UESTCC 22.0144	OP956043	OP955974	OP955999	OP961440	-
*P. circinata*	CBS 263.37	-	MW810265	MH867413	MW735660	-
***P. citlaltepetlensis***	**ENCB 140251 = IOM 325319.1**	**-**	**MH890645**	**MT625978**	**-**	**-**
*P. citlaltepetlensis*	IOM 325319.2	-	MT649221	MT649216	-	-
*P. cookei*	MFLUCC 17–1399	-	MG333490	MG333493	MG438279	-
*P. cookei*	MFLUCC 17–1679	-	-	MG333492	MG438278	-
*P. cookei*	UESTCC 22.0134	OP956037	OP955968	OP955993	-	OP961459
*P. cortaderiae*	MFLUCC 15–0451	KX986346	KX965734	KX954403	KY429208	-
*P. cortaderiae*	MFLUCC 15–0453 = ICMP 21429	-	KX965733	KX954402	KY320574	-
***P. cortaderiae***	**MFLUCC 15–0457 = ICMP 21414**	**KX986345**	**KX965732**	**KX954401**	**KY310703**	**-**
***P. cynodontis***	**CGMCC 3.23927 = UESTCC 22.0127**	**OP909920**	**OP909925**	**OP909921**	**OP961434**	**OP961460**
***P. cyperacearum***	**CPC 32138 = CBS 144434**	**-**	**MH327815**	**MH327851**	**-**	**-**
***P. delonicis***	**MFLUCC 17–2584 = KUMCC 18–0275**	**MK347832**	**-**	**MK347941**	**MK360071**	**MK434901**
***P. didymosporum***	**MFLU 15–0058**	**KP761738**	**KP761734**	**KP761731**	**KP761728**	**KP761721**
*P. digitata*	CBS 510.77	AB797271	LC014584	AB807561	AB808537	-
***P. elaeidis***	**MFLUCC 17–0087**	**MH108551**	**MG742713**	**MH108552**	**-**	**-**
***P. epilithographicola***	**CBS 144017**	**-**	**NR_157477**	**-**	**-**	**-**
*P. epilithographicola*	MFLUCC 21–0153	OL606144	OL753687	OL606155	OL912948	-
***P. festucae***	**CGMCC 3.23929 = UESTCC 22.0128**	**OP956042**	**OP955973**	**OP955998**	**OP961439**	**OP961463**
*P. genistae*	CBS 322.79	-	MH861213	MH872980	-	-
***P. homothallica***	**CBS 139698 = JCM 13100 = MAFF 239610 = KT916 = HHUF 29105**	**AB797275**	**AB809645**	**NG_059397**	**AB808541**	**-**
*P. igniaria*	CBS 298.66	-	MH858798	MH870438	-	-
*P. igniaria*	CBS 583.66	-	MH858888	MH870553	-	-
***P. imperatae***	**CGMCC 3.23931 = UESTCC 22.0129**	**OP956053**	**OP955984**	**OP956009**	**OP961450**	**OP961467**
*P. imperatae*	UESTCC 22.0145	OP956048	OP955979	OP956004	OP961445	-
*P. imperatae*	UESTCC 22.0146	OP956052	OP955983	OP956008	OP961449	-
*P. lateralis*	CBS 292.36	-	MH855804	MH867311	-	-
*P. macrospinosa*	CBS 135663 = CPC 22898	KP184080	KP183999	KP184038	-	-
*P. minutissima*	MFLUCC 15–0245	-	KY794703	KY794707	-	-
***P. neobrittanica***	**CPC 37903 = CBS 146062**	**-**	**MN562149**	**MN567656**	**-**	**-**
***P. palmicola***	**MFLUCC14–0400**	**MN648319**	**-**	**MN648327**	**MN821070**	**-**
***P. penniseti***	**CGMCC 3.23928 = UESTCC 22.0130**	**OP956040**	**OP955971**	**OP955996**	**OP961437**	**OP961462**
***P. prolifica***	**CBS 209.64**	**-**	**NR_160097**	**MH870050**	**-**	**-**
*P. pseudobyssoides*	DLUCC 0850	-	MG333491	MG333494	MG438280	-
*P. pseudobyssoides*	H4151 = MAFF 243868	AB797278	LC014587	AB807568	AB808544	-
*P. pseudobyssoides*	KUMCC 20–0263	MW444853	MW444851	MW444852	MW460894	-
*P. pseudobyssoides*	MAFF 243874 = TS102 = HHUF 28257 = H 4790	AB797270	LC014588	AB807560	AB808536	-
*P. pseudobyssoides*	UESTCC 22.0135	OP956034	OP955965	OP955990	OP961431	OP961456
*P. pseudobyssoides*	UESTCC 22.0147	OP956044	OP955975	OP956000	OP961441	-
***P. pseudodigitata***	**CBS 139699 = JCM 13166 = MAFF 239676 = KT 1395 = HHUF 29730**	**AB797274**	**LC014591**	**AB807564**	**AB808540**	**-**
*P. pseudodigitata*	JCM 13164 = MAFF 239674 = KT644	AB797272	LC014589	AB807562	AB808538	-
*P. pseudodigitata*	JCM 13165 = MAFF 239675 = KT 1195A	AB797273	LC014590	AB807563	AB808539	-
***P. sahariana***	**CBS 320.79**	**–**	**MH861211**	**MH872978**	**-**	**-**
***P. salina***	**GJ374 = MFLU 19–1235**	**MN017912**	**MN047086**	**MN017846**	**-**	**-**
***P. spodiopogonis***	**CGMCC 3.23932 = UESTCC 22.0131**	**OP956032**	**OP955963**	**OP955988**	**OP961429**	**OP961454**
***P. submersa***	**MFLUCC 16–1098**	**-**	**KY794702**	**KY794706**	**KY814761**	**-**
***P. thailandica***	**MFLUCC 17–0065**	**KY753889**	**KY753887**	**KY753888**	**–**	**-**
***P. thysanolaenae***	**KUMCC 20–0262**	**NG_081407**	**MW442967**	**MW444850**	**MW460896**	**-**
***P. variicolor***	**CBS 120374 = SACCR 64**	**-**	**DQ336713**	**-**	**-**	**-**
***P. verrucosa***	**MFLUCC 17–2158**	**MT226686**	**MT310617**	**MT214572**	**MT394631**	**-**
*P. verrucosa*	UESTCC 22.0136	OP956035	OP955966	OP955991	OP961432	OP961457
*P. verrucosa*	UESTCC 22.0148	OP956039	OP955970	OP955995	OP961436	-
*P. verrucosa*	UESTCC 22.0149	OP956045	OP955976	OP956001	OP961442	OP961464
*P. verrucosa*	UESTCC 22.0150	OP956049	OP955980	OP956005	OP961446	-
*P. verrucosa*	UESTCC 22.0151	OP956051	OP955982	OP956007	OP961448	-

The newly generated sequences are indicated in red, and the ex-type strains are in bold. Missing sequences are indicated by “-”.

## Data Availability

All sequence data are available in NCBI GenBank following the accession numbers in the manuscript.
